# Employment status of person with disability in Government Departments in Khyber Pakhtunkhwa Pakistan

**DOI:** 10.12669/pjms.35.5.239

**Published:** 2019

**Authors:** Aatik Arsh, Haider Darain, Amir Zeb, Sana Ullah, Irfan Ullah, Syed Muhammad Ilyas

**Affiliations:** 1 Aatik Arsh, DPT, MSPT. Physical Therapist, Paraplegic Centre Peshawar, Pakistan; 2 Haider Darain BSPT, MSc, PhD. Assistant Professor Khyber Medical University Peshawar, Pakistan; 3 Amir Zeb, BSPT, PPDPT, MSPT. Senior Physical Therapist, Paraplegic Centre Peshawar, Pakistan; 4 Sana Ullah, MS software Engineering. Occupational therapy assistant Paraplegic Centre Peshawar, Pakistan; 5 Irfan Ullah, BS Electronics. General Secretary Friends of Paraplegics Peshawar, Pakistan; 6 Syed Muhammad Ilyas, BSPT. Chief Executive Officer, Paraplegic Centre Peshawar, Pakistan

**Keywords:** Disability, Employment, Health, Law, Policy, Pakistan, Society

## Abstract

**Objective:**

The objective of the study was to determine whether anticipated number of person with disabilities was employed in different government departments of Khyber Pakhtunkhwa (KPK), Pakistan.

**Methods:**

Using across sectional survey, data from 16 different departments of provincial government of KPK was collected by volunteers of ‘Friends of Paraplegics.

**Results:**

Out of total 1, 71,137 Govt. employees, only 1151 (0.67%) were person with disabilities. None of the included departments fulfilled 2% allocated job quota for person with disabilities. Majority of the employees included in study were from Elementary & Secondary Education Department (n=140345) and Agriculture, Livestock & Cooperation Department (n=14315). The number of person with disabilities in these departments were 960 (0.68%) and 68 (0.48%) respectively. The highest percentage of person with disabilities were working in Higher Education Archives & Libraries Department (1.65%)followed by Law, Parliamentary Affairs & Human Rights Department (1.42%), Planning & Development Department (1.39%) and Administration & Establishment Department (1.16%).

**Conclusion:**

It is concluded that a small number of person with disabilities are employed in different Govt. Departments. Moreover, these departments has not ensured providing 2% job quota for person with disabilities.

## INTRODUCTION

Disability is associated with incredible human sufferings all over the globe and Persons With Disabilities (PWDs) have remained marginalized part of society in all countries in general and in the developing countries in particular.^1^ According to World Health Organization (WHO), a large proportion of the world’s population (almost 15%) live with some sort of disability.^2^ However, due to unavailability of reliable data in some countries the reported number of PWDs is not according the anticipated number. Pakistan is one those countries where the reported number of PWDs has not reported according to the anticipated figures reported by WHO (2.49% of the population reported in census 1998).^3^ It has been suggested that the estimated number of PWDs in the country may be around 10%.^4^ Data from the country show that out of the country population, only 136,928 PWD’s has been issued national identity cards and may be regarded registered PWDs in the country.^5^

The reported number of PWDs in developing countries is high compared to the number reported in developing countries. The presence of some of the modifiable factors including poverty, illiteracy and continuous exposure to armed conflicts in these countries might be associated with the bigger number of PWDs in these countries.^6^ In Pakistan, majority of PWD’s live a dependent life and in most cases, their family members are responsible for the support.^7^ Dependency is one of the main cause due to which PWD’s are often disregarded and are mistreated in society.^8^ In order to protect rights of PWD’s and to make them functional members of society by providing them employment opportunities, Government of Pakistan provide many constitutional and legal provisions to PWD’s living in Pakistan. The most important national policy in this regard is fixed job quota for PWD’s in both government and private sector. In 1981, ex-president of Pakistan Zia-ul-Haq, allocated 1% job quota for PWD’s through ordinance “Disabled Persons (Employment and Rehabilitation) Ordinance (DPO-1981)” while in 1998Prime minister Pakistan Nawaz Sharif increased PWD’s job quota from 1% to 2%.^9^,^10^ The present job quota for PWD’s in all provinces of Pakistan is 2% except Punjab where the quota was raised to 3% in 2015.^5^

Despite a number of national policies regarding legal rights of PWD’s in the country, PWD’s are neglected and ignored part of the community.^4^,^7^,^11^ Despite the fact single centred epidemiological surveys carried out about the epidemiology of spinal cord studies in the country might be sound in the literature, yet, no official updated statistics regarding PWD’s in the county can be found. Moreover, financial status, living standards and source of income of PWD’s in the country still remained unclear. This might indicate the level of commitment of official agencies towards the betterment of PWDs in the country. Moreover, this area in the country has not received robust attention and according to authors’ knowledge, limited data might be found about the situation of PWDs the country.^7^,^11^ An addition, no scholarly work was available in the field of right of employment of PWD’s in Pakistan. Therefore, this large scale survey was designed to determine number of PWD’s employed in different government departments in Khyber Pakhtunkhwa, Pakistan. This will helpful to evaluate whether government departments are following 2% fixed job quota for PWD’s.

## METHODS

A cross sectional study was conducted in Khyber Pakhtunkhwa, Pakistan, using quantitative approach. Ethical approval was obtained from Ethics committee of Paraplegic Centre Peshawar. The study duration was one year, from March 2016 to March 2017. Volunteers of Friends of Paraplegics collected data of total employees and PWD’s working in different government departments of KPK. Data was collected from 16 provincial government departments. The data of total number of employees and number of PWD’s employees were transferred to excel sheet. Number of PWD’s required as per 2% quota was calculated by using formula (2%*total number of employees in a department) while number of PWD’s need to be hired were calculated by using formula (Number of PWD’s required as per 2% quota minus Number of PWD’s employee).

## RESULTS

Data of 171137 employees working in 16 different government departments of KPK was collected. Results showed that out of total 171137 employees, only 1151 (0.673%) employees were PWD’s. When 2% quota was calculated, it was revealed that 2% of 171137 employees is 3423.

Out of 16 department’s form which data was collected, no single government department in KPK fulfill 2% job quota for PWD’s. The majority of employees included in study were from Elementary & secondary education department (n=140345) and Agriculture, Livestock & Cooperation department (n=14315) but number of PWD’s in these departments were 960 (0.684%) and 68 (0.475%) respectively. The number of PWD’s to be hired in Elementary & secondary education department and Agriculture, Livestock & Cooperation department to fulfill 2% job quota is 1847 and 218 respectively. (Table-I)

**Table I:** Table showing number of PWDs working in various departments of Khyber Pakhtunkhwa Pakistan.

Department	Total employee	Number of PWD’s employee
Administration and Establishment department	2064	24 (1.16%)
Agriculture, Livestock and Cooperation department	14315	68 (0.48%)
Auqaf, Hajj, Religious & Minority Affairs department	427	1 (0.23%)
Elementary and secondary education department	140345	960 (0.68%)
Finance department	1836	3 (0.16%)
Forestry, Environment And Wildlife department	73	0 (0%)
Health department	2101	17 (0.81%)
Higher Education Archives & Libraries department	2369	39 (1.65%)
Home & Tribal Affairs department	260	2 (0.77%)
Information & Public Relations department	125	0 (0%)
Irrigation department	51	0 (0%)
Labor department	1030	7 (0.68%)
Law, Parliamentary Affairs and Human Rights department	1271	18 (1.42%)
Planning & Development department	144	2 (1.39%)
Population welfare department	4347	10 (0.23%)
Transport department	379	0 (0%)

Total	171137	1151 (0.67%)

The highest percentage of PWD’s were in Higher Education Archives & Libraries department (1.646%) followed by Law, Parliamentary Affairs & Human Rights department(1.416%), Planning & Development department (1.389%) and Administration & Establishment department (1.163%). All other 12 departments were having less that 1% PWD’s. (Table-I)

## DISCUSSION

Unequal employment rates between PWD’s and non-disabled people is reported in many countries.^12^ Several policies on national and international level have been developed in recent decades to address this issue of unequal employment rates ofPWD’s.^12^-^14^ Disability quota system is example of one such policy which is implemented in many countries in order to fill the employment gap between PWD’s and able-bodied people. Many Asian countries including Pakistan have implemented quota system for PWD’s in order to provide employment opportunities for them.^15^,^16^ Nevertheless, many studies reported ambiguous results regarding effectiveness of these disability quota system.^12^,^17^,^18^ Similarly, a previous study conducted in Pakistan also reported that majority of PWD’s in Pakistan were having view that quota system is useless as it is not followed in any government or private organization.^16^ Little information was available regarding PWD’s employed in government departments in Pakistan and consequently no information was available whether different government departments are following 2% fixed quota system for PWD’s or not. To the author’s the current study was the first study in country, which reported number of PWD’s working in different government departments in KPK, Pakistan and provide statistical information that number of PWD employees are negligible in government departments and almost all government departments failed to achieve 2% employment of PWD’s.

Govt. of Pakistan took many steps to provide employment opportunities to PWD’s. For example, National policy for PWD’s 2002 clearly mentioned that “Pakistan joined the select group of countries, which has not only ratified ILO Convention 159, but have also taken active legal steps to introduce legislation which lays down quota for the employment of persons with disabilities”. It is further added that, “The penalty clauses will also be amended to make its implementation more effective”.^19^ Section 11 of The Disabled Persons (Employment and Rehabilitation) Ordinance, 1981 also emphasizes that “An establishment which does not employ a disabled person shall pay in to the Funds each month the sum of money it would have paid as salary or wages to a disabled person had he been employed”. Keeping in view the fact, that 2% quota for PWD’s is not following in Pakistan, Aisha Syed, member of National Assembly of Pakistan presented “The Disabled Persons (Employment) and Rehabilitation (Amendment) Act 2015” in National Assembly in order to strengthen the rights of PWD’s in Pakistan. The said bill emphasizes that serious actions to be taken against departments which ignores 2% quota of PWD’s. After 18^th^ Amendment to the Constitution of Pakistan in 2010 the disability and all issues related to it becomes provincial subject and now provincial government is responsible for issues related to PWD’s.^20^

KPK, one of the neglected province of Pakistan, where the exact statistics regarding PWD’s is not available yet it is estimated that around 400,000 PWD’s are living in province.^21^ The ground reality is that, this figure is just assumption and because of under reporting of disability there are thousands of PWD’s which are not registered and the fact is that, the exact figure of PWD’s is much higher than this. This shows that we even don’t know exact number of PWD’s, so how can we plan to address issues related to PWD’s. One of the previous study also reported that Scarcity of reliable disability epidemiologic data is one of the major barrier in addressing issues related to PWD’s in Pakistan.^11^ Negative attitude and superstitious beliefs towards PWD’s is reported by many research studies and is evident throughout history. Though many studies conducted in Pakistan, reported social rejection of PWD’s in country and truth is that problems of PWD’s are much more than that yet due to religious affiliations, general population living in Pakistan demonstrate strong afflation’s towards PWD’s. Islamic teachings focuses on the fact that it is the responsibility of government as well as of society to support PWD’s and provide equivalent opportunities of learning and employment to PWD’s.^19^,^22^

The facts and figures revealed by results of current study are alarming. The current study collected data of 1,71,137 government employees, out of which only 1151 (0.673%) employees were PWD’s. In Pakistan, due to lack of facilities, mostly PWD’s are unable to continue their education and therefore unable to get higher degrees which is main hindrance in getting high level posts. But the truth is that in majority of cases even highly qualified PWD’s are deprived of job opportunities in government departments. Majority of people living in community are unaware of the capabilities of PWD’s and that’s why government officers are usually hesitant to employ PWD’s as they have reservations that PWD’s might not be able to carry out their duties efficiently. There are reports that in Pakistan, that due to the fear that hiring of PWD’s may affect output, employers prefer to pay fine rather than hiring PWD’s.^7^ Another important barrier to employment of PWD’s in Pakistan is that majority of places including government departments in country are not wheel chair accessible. Similarly, public transport is also not accessible to PWD’s.

The current study also demonstrate that, although laws are present regarding 2% quota for PWD’s, however practically PWD’s are deprived of their right of 2% seats in government departments. Results showed that none of the 16 departments from which data was collected for current study followed 2% job quota for PWD’s. Only 4 departments were having greater than 1% PWD’s while 12 were having less than 1% PWD’s. It is clearly mentioned in Civil Establishment Code (ESTA code) KPK that “two percent of all posts in each basic pay scale to be filled in by initial recruitment shall be reserved for disabled candidates”.^23^ However analysis of data of current study showed that almost all provincial departments are not following the policy of fixing 2% job quota for PWD’s. The low employment rate of PWD’s is not only due to negligence of government departments but also due to the fact that majority of PWD’s are unaware of their legal rights. One of the previous study reported that more than half of PWD’s living in Pakistan are even not aware regarding quota system for PWD’s, which shows the negligence of society and education system that no one guide them about their legal rights.^16^

Due to time limitation and limited resources current study had some limitations. We had collected data from only 16 government departments while there are about 34 government departments in KPK. Similarly, we had collected data of 1,71,137 government employees numbers, which is almost equal to number of government employees working in only one department, that is Elementary and secondary education department, in which there are about 1,68,000 employees. Another limitation is that we collected data only from government departments and missed thousands of employees working in private sector. Regardless of the fact that number of employees and number of departments included in current study are limited as compared to total number of employees and total number of KPK government departments, yet it is one of the first study, which reported valuable information regarding PWD’s working in different government departments of KPK.

## CONCLUSIONS

The results of current study showed that number of PWD’s working in different government departments in KPK, Pakistan is negligible as compared to non-disabled people. Secondly, it was revealed that none of the government department is following 2% job quota for PWD’s. On the basis of facts and figures presented in current study it is recommended that government should develop strict strategies to implement 2% fixed job quota for PWD’s. Large trials are needed to be carried out to find out number of PWD’s working in public and private sector in different provinces of Pakistan and to determine barriers to employment of PWD’s in country.
